# MRI imaging of soft tissue tumours of the foot and ankle

**DOI:** 10.1186/s13244-019-0749-z

**Published:** 2019-06-03

**Authors:** Peter Hughes, Rhian Miranda, Anthony J. Doyle

**Affiliations:** Department of Radiology, Auckland City Hospital, Auckland, New Zealand

**Keywords:** Magnetic resonance imaging, Foot, Ankle, Neoplasms, Differential diagnosis, Soft tissue

## Abstract

The majority of soft tissue lesions in the foot and ankle are benign. The aim of this review is to provide the reader with a comprehensive overview of the magnetic resonance imaging (MRI) characteristics of the most common benign and malignant soft tissue neoplasms which occur around the foot and ankle. This should enable the reader to formulate a reasonable differential diagnosis and, most importantly, to recognise those rare aggressive lesions that require further assessment and tissue biopsy.

## Key points

- Soft tissue masses of the foot and ankle are relatively infrequent and may pose a diagnostic dilemma
- Although much less frequent, malignant neoplastic processes can mimic benign lesions and may be difficult to differentiate
- Radiologists should have a thorough knowledge of the imaging characteristics of these lesions to advise on appropriate surgical management.

## Introduction

Soft tissue masses of the foot and ankle are relatively infrequent and may pose a diagnostic dilemma [1]. Ultrasound (US) evaluation of soft tissue lesions is useful in the initial triaging of soft tissue lesions. US can demonstrate the cystic nature of some benign conditions such as ganglions or synovial cysts and may also be diagnostic in some other benign lesions such as superficial lipoma (if stable over 6 months), Morton neuroma, foreign body granuloma and plantar fibromatosis [2]. However, it should be used with caution as it may be difficult to interpret and can be associated with a delay in diagnosis of malignant lesions [3]. Magnetic resonance imaging (MRI) evaluation should follow on from US in any case where there is a reasonable chance of malignancy, where a lesion is incompletely evaluated, and in any lesion which is > 5 cm, crosses or lies deep to the superficial fascia or occurs at a site of previous resection [2].

MRI provides excellent anatomical detail and allows for soft tissue characterisation which plays an important role in formulating a differential diagnosis. It allows for local staging and description of the relationship of a lesion to adjacent anatomical structures (e.g. fascia, bone, muscle, neurovascular structures). Furthermore, advanced MRI techniques may aid in soft tissue characterisation. Proton magnetic resonance spectroscopy (PMRS) may demonstrate an elevated choline peak within a soft tissue lesion, indicative of the high cellular turnover seen in malignancy [4, 5]. Susceptibility weighted imaging (SWI) allows for easier detection of low signal haemosiderin, melanin and calcification within a lesion, as found for example in metastatic melanoma [4]. Lower apparent diffusion coefficient (ADC) scores on diffusion-weighted imaging (DWI) are generally seen in malignant lesions compared with benign lesions. ADC values may also help in differentiating necrotic malignant lesions from abscesses. Greater diffusion restriction is seen within the necrotic core of an abscess compared with necrotic tumours, which tend to have greater diffusion restriction in the solid tumour components [4, 5]. Myxoid tumours have been shown to have a higher ADC value than non-myxoid lesions [4–6]. Dynamic contrast-enhanced (DCE) perfusion imaging may aid in differentiating benign from malignant lesions based on patterns of contrast enhancement and washout. In addition, it can assess for viable, enhancing tumour to guide biopsy and may aid in early detection of residual/recurrent tumour post resection [4, 5]. It should be kept in mind that considerable overlap between benign and malignant lesions has been found with all of these techniques. Radiomic analysis of MR images has been shown to have some prognostic value in soft tissue sarcoma but is not yet a mature technique [7, 8].

Plain radiography is almost always useful as an adjunct to US. It allows characterisation of mineralisation within a lesion and can be useful to assess for the presence of phleboliths. It is also useful to assess the extent of underlying bone and joint involvement, which can both suggest an aetiology and have important implications for surgical treatment. Computed tomography (CT) can be very helpful in the foot and ankle because of the complex anatomy and overlapping structures.

Fluorine-deoxyglucose positron emission tomography/computed tomography (FDG PET/CT) is not usually indicated in the initial evaluation of a soft tissue lesion. There is however a correlation between standardised uptake value (SUV) max and histologic subtype in soft tissue sarcomas with high grade tumours demonstrating a higher SUV max value. It has also been shown to be very sensitive in assessing for distant metastatic disease, in directing a target for biopsy (avoiding non-diagnostic samples secondary to tumour necrosis) and in evaluating treatment response [9, 10].

A variety of neoplastic and non-neoplastic lesions may occur around the foot and ankle, the vast majority of which are benign. The most common malignant lesions are synovial sarcoma and pleomorphic undifferentiated sarcoma [11]. The most common benign lesions include plantar fibromatosis, lipoma, peripheral nerve sheath tumours and giant cell tumours of the tendon sheath [12]. Table 1 shows the breakdown of the most common lesions by age group. Malignant neoplastic processes can mimic benign lesions and may be difficult to differentiate [3, 13]. This highlights the importance of a thorough knowledge of the imaging characteristics of these lesions. The non-neoplastic “pseudotumoural” lesions have been covered in this journal previously by Van Hul et al. [13]. This review focuses on the MRI characteristics of the most common neoplastic lesions of the foot and ankle, both benign and malignant.

**Table 1:** Table showing the most common benign and malignant lesions of the foot and ankle subdivided by age group as described by Kransdorf [7, 8]

Age group	Benign lesions	%	Malignant lesions	%
0–5 years	• Granuloma annulare • Fibromatosis • Haemangioma	30 25 11	• Fibrosarcoma • DFSP • MPNST • Rhabdomyosarcoma	45 18 18 18
6–15 years	• Fibromatosis • Granuloma annulare • Haemangioma	23 13 13	• Synovial sarcoma • DFSP • Rhabdomyosarcoma	21 17 9
16–25 years	• Fibromatosis • GCTTS • Granuloma annulare • Fibrous histiocytoma	22 14 12 12	• Synovial sarcoma • Clear cell sarcoma • Fibrosarcoma • DFSP	30 11 8 8
26–45 years	• Fibromatosis • Fibrous histiocytoma • PNST • GCCTS	25 13 11 9	• Synovial sarcoma • Clear cell sarcoma • Pleomorphic undifferentiated sarcoma	26 13 13
46–65 years	• Fibromatosis • Fibrous histiocytoma • Lipoma • PNST	25 13 11 8	• Pleomorphic undifferentiated sarcoma • Synovial sarcoma • Leiomyosarcoma • Kaposi’s sarcoma	25 17 12 9
66 and over	• Fibromatosis • PNST • Fibrous histiocytoma • Chondroma	14 13 11 9	• Kaposi’s sarcoma • Pleomorphic undifferentiated sarcoma • Leiomyosarcoma	37 19 15

## Benign lesions

### Plantar fibromatosis

The plantar fascia is a connective tissue structure which helps to maintain the longitudinal arch of the foot. It extends from the medial tubercle of the calcaneus, covering the flexor digitorum brevis and dividing into branches which insert into the metatarsophalangeal joints. There are central and lateral bands, with a thinner medial band arising from the mid portion of the central band.

Plantar fibromatosis (Ledderhose disease) is a common benign proliferative disorder of the plantar fascia composed of fibroblasts and collagen fibres. It occurs predominantly in the 4th–6th decade with males being more commonly affected than females. Bilateral disease is present in up to 20–50% of cases and multiple nodules are present in 33% cases [14–22]. It is more common in those with diabetes, epilepsy and alcohol excess [19]. There is an association with other fibrotic diseases such as palmar fibromatosis (Dupuytren’s disease) which co-exists in 40% and penile fibromatosis (Peyronnie’s disease) [14, 15, 18]. It more often affects the distal two-thirds, though proximal nodules also occur [16, 17]. It manifests as a discretely palpable mass which is not usually painful unless very large.

**Fig. 1:**
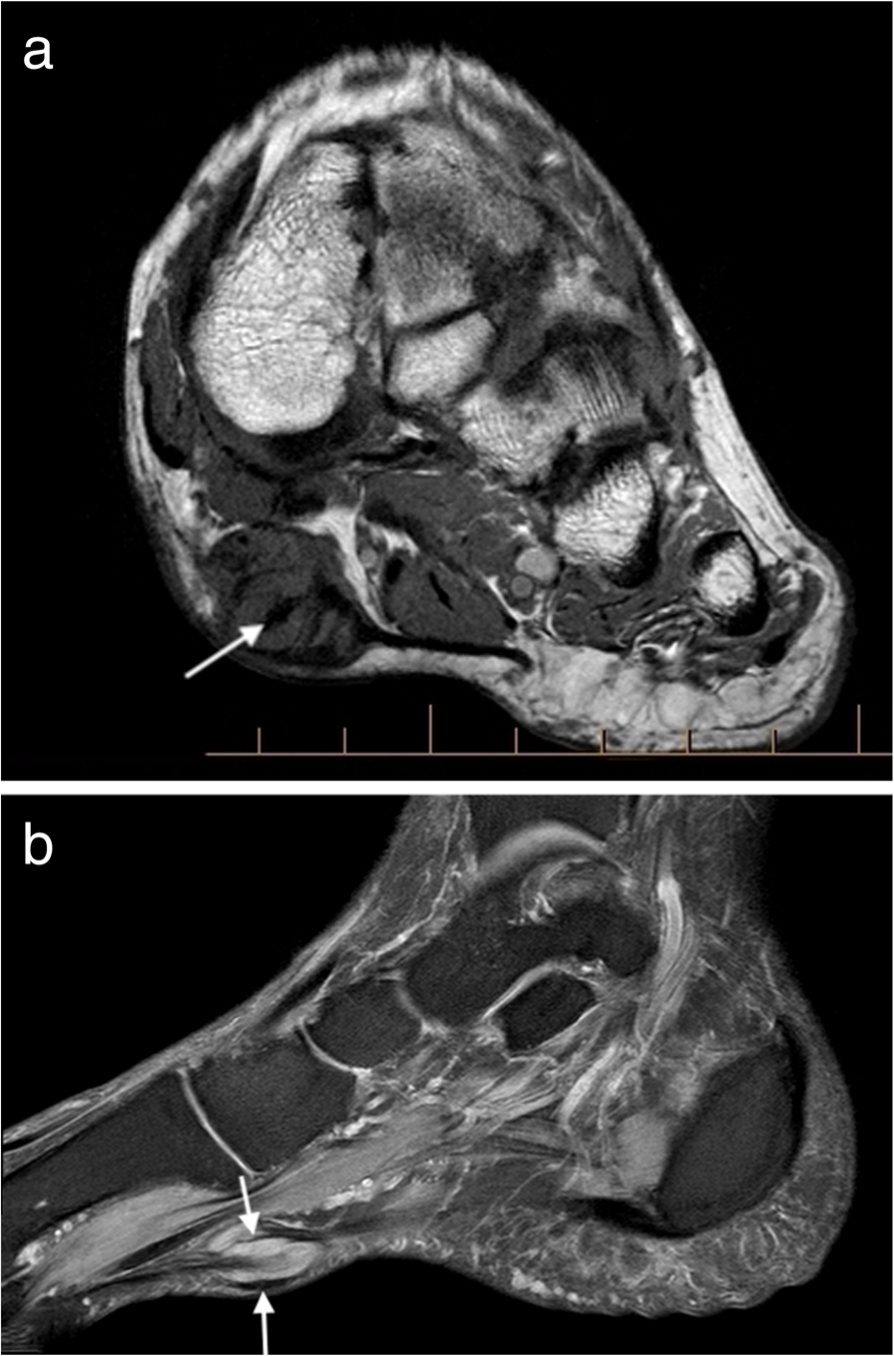
Plantar fibroma. **a** Sagittal T2 fat suppressed (T2FS) and (**b**) coronal T1-weighted image of the foot demonstrating a heterogeneous mass centred on the plantar fascia, distinct from the underlying musculature. The characteristic low signal linear bands are shown (arrows)

Ultrasound alone may be sufficient for diagnosis in plantar fibromatosis. It appears as a fusiform hypoechoic or heterogeneous mass arising from the plantar fascia [16, 19]. Similarly, on MRI, it is seen as a fusiform mass arising from the plantar fascia, more often medial than lateral [16, 17]. It is usually heterogeneous in signal intensity, hypointense to skeletal muscle on T1-weighted (T1W) imaging and iso- to hyperintense to skeletal muscle on T2-weighted (T2W) imaging (Fig. 1) [14–21]. Early lesions are more cellular and tend to be more hyperintense on T2W imaging, whereas more mature lesions tend to be more fibrotic with low signal on T2W [15]. The deep margin is often not well demarcated from the underlying muscle, but the superficial margin is usually clearly defined. It demonstrates variable enhancement on post contrast imaging, which is not generally required for the diagnosis [14, 17].

### Deep fibromatosis

Deep fibromatosis is a rare, locally aggressive lesion composed of fibroblasts embedded in a collagen matrix with a peak age of onset between 25 and 40 years. It is slightly more common in females [14, 15]. Multiple lesions may occur in the same extremity. It usually presents as a painless mass but may have local mass effect resulting in impingement or neural compression. Both plantar and deep fibromatosis are characterised by an infiltrative growth pattern and have a tendency toward local recurrence post surgical resection, the latter being more aggressive. Fibromatosis does not metastasise [15].

**Fig. 2:**
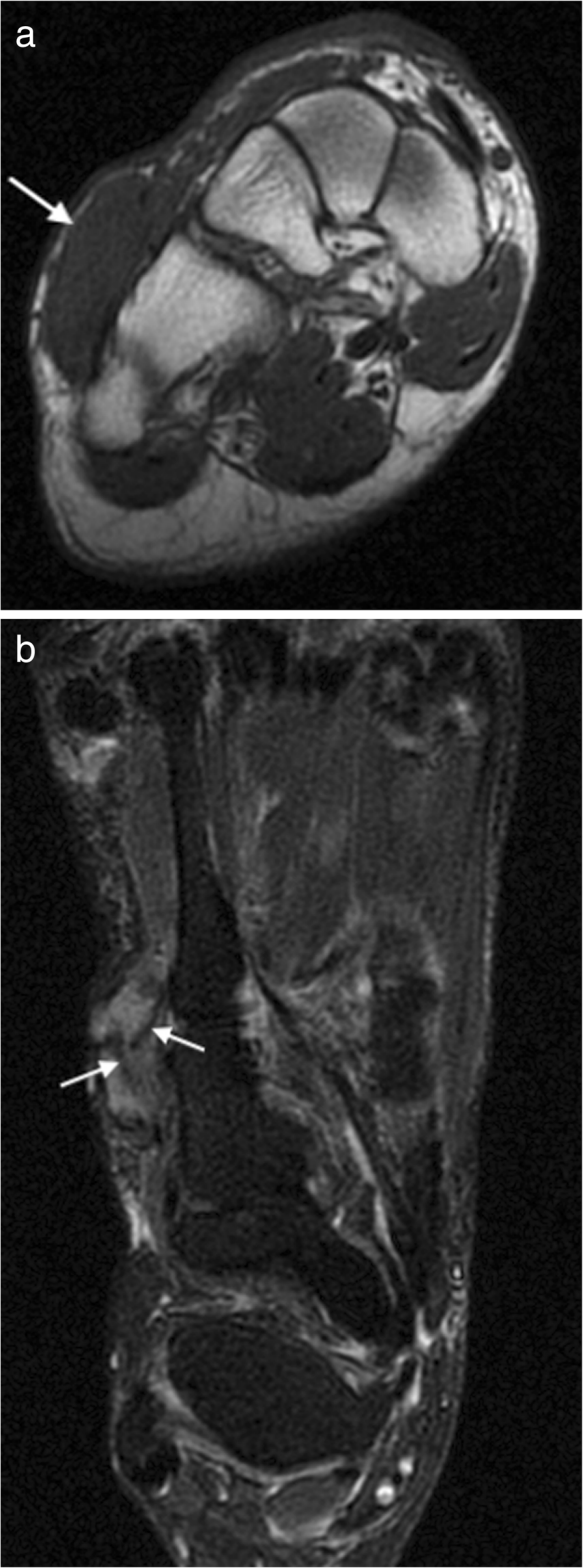
Deep fibromatosis. Axial T1W (**a**) and T2FS (**b**) images showing a mass on the dorsum of the foot. The mass is isointense to muscle on T1W. On T2FS, the mass is hyperintense with linear low signal collagen bands (arrows)

On MRI, it has a variable appearance, reflecting the variable composition of the lesion (Fig. 2). The mass tends to grow along fascial planes and may cause displacement or encasement of adjacent tendons, ligaments and muscles. This tendency to grow along the fascia or aponeurosis is known as the “fascial tail sign” and has been described in up to 80% cases, though is not specific to this condition [23]. There may be pressure erosion on the adjacent bone. The lesion is generally isointense to muscle on T1W and iso- to hyperintense on T2W with enhancement on post contrast imaging. Band-like foci of low signal on all sequences has been described as a characteristic finding. These low signal collagen bands show little or no enhancement on post contrast imaging, a finding also seen in old, mature or radiation therapy-treated lesions [14, 15, 18, 24].

Oka et al. have suggested that diffusion-weighted imaging may help to differentiate desmoid tumours from malignant soft tissue tumours, with fibromatosis showing a higher apparent diffusion coefficient (ADC) than malignant tumours [25]. Larger studies are required to validate this technique.

### Haemangioma

Lesions commonly referred to as haemangiomas belong to the spectrum of vascular anomalies. In an effort to standardise nomenclature across this heterogeneous group of lesions, the International Society for the Study of Vascular Anomalies (ISSVA) published a revised classification system which divides vascular lesions into neoplastic tumours and non-neoplastic malformations. Vascular malformations are further subdivided into capillary, lymphatic, venous, arteriovenous and combined subtypes. There are separate categories for lesions associated with major named vessels, lesions associated with other anomalies and provisionally unclassified vascular anomalies [26].

Lesions around the foot and ankle will generally fall into the vascular malformation category. They may be superficial or deep and may be associated with fat, fibrous tissue and/or smooth muscle, often adapting their shape to adjacent structures. They account for 7% of benign soft tissue lesions in the foot and ankle [17]. They most commonly present before 30 years of age and are often asymptomatic but may present with pain after exercise secondary to a local vascular steal phenomenon [13]. A bluish skin discolouration is a classic examination finding. Radiographs are helpful to demonstrate phleboliths and may show associated periosteal reaction or cortical thickening [1].

**Fig. 3:**
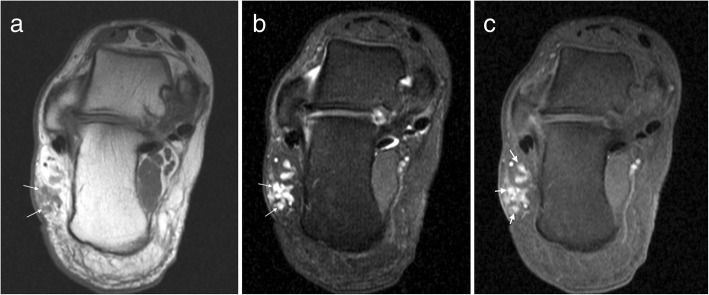
Vascular malformation. **a** Axial T1W and (**b**) proton density with fat suppression (PDFS) images of the ankle demonstrating a low flow vascular malformation overlying the lateral aspect of the calcaneus with delayed multinodular enhancement (arrows) on the post contrast T1FS image (**c**)

On MRI, vascular malformations may be well or poorly defined lesions with little mass effect for their size (Fig. 3). They may have high signal on T1W, reflective of fat content or internal haemorrhage. The majority are slow flow lesions which appear T2 hyperintense and may demonstrate fluid-fluid levels. High flow lesions (arteriovenous malformations) demonstrate serpiginous flow-voids. Phleboliths may be seen as foci of low signal on all sequences and should be correlated with plain film radiography. High flow lesions generally demonstrate avid enhancement but may occasionally show delayed enhancement. The pattern of enhancement reflects the composition of the lesion and the flow characteristics of the involved vessel subtype. Atrophy in the adjacent muscle may be due to vascular steal and perilesional haemorrhage may also be present [14, 15, 17, 27].

### Angiomyoma

Also known as vascular leiomyoma or angioleiomyoma, this is a rare subcutaneous leiomyoma, most often occurring in females in the 4th–6th decade. It has a predilection for the lower extremity, most commonly around the foot and ankle [14, 15, 28]. They are usually small (< 2 cm) slow growing, painful, round or ovoid tumours arising from small vessels in the deep dermis or subcutaneous fat and are composed of small vascular channels in smooth muscle [14, 15, 28].

**Fig. 4:**
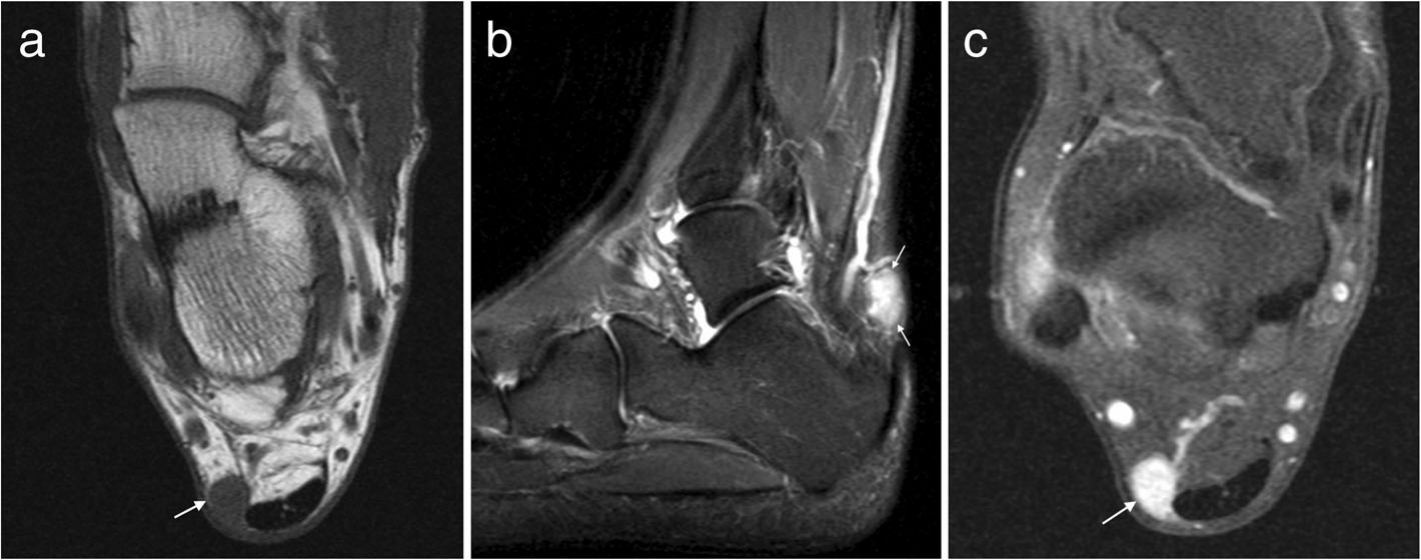
Angiomyoma. Axial T1W (**a**), sagittal T2FS (**b**) and post contrast T1FS (**c**) images of the ankle showing a well-defined lesion adjacent to the Achilles tendon which appears isointense on T1W, hyperintense on T2W and avidly enhances on postcontrast imaging. Note the capsule visible on T2FS (arrows)

On MRI, they tend to have a low signal fibrous capsule. Rarely, they may cause pressure erosion of adjacent bones. They tend to be slightly hyperintense on T1-weighted imaging and heterogeneous on T2-weighted imaging with marked enhancement within the vascular component (Fig. 4) [14, 15, 28].

### Lipoma

**Fig. 5:**
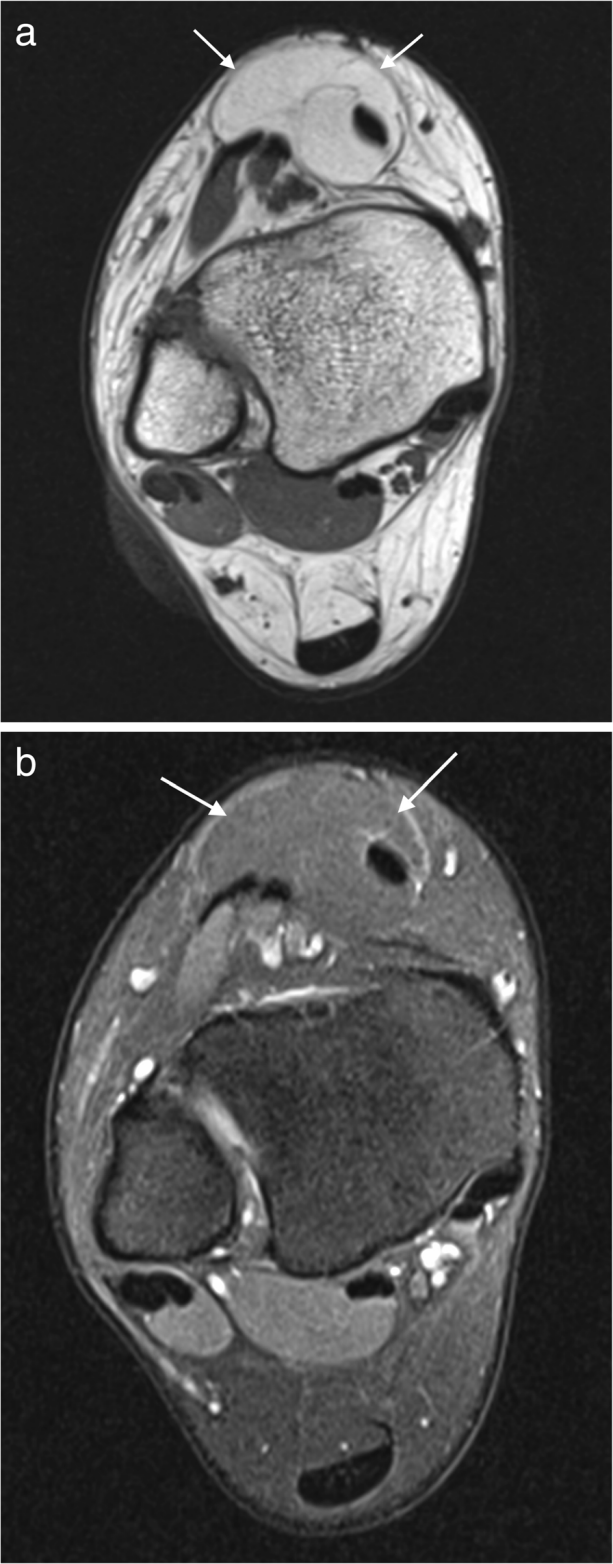
Lipoma. Axial T1W (**a**) and PDFS (**b**) images of the ankle demonstrating an encapsulated lesion (arrows). The lesion is isointense to fat on T1 with uniform loss of signal on PDFS consistent with a lipoma

These are benign encapsulated tumours composed of mature adipocytes. Lipomas are the most common soft tissue tumour in the body, but uncommon around the foot and ankle [14, 16, 18, 19]. On MRI, lipomas follow the signal of subcutaneous fat (Fig. 5), being hyperintense on both T1- and T2 weighted imaging and demonstrating uniform loss of signal on fat suppression sequences [14–18, 27]. Many lipomas will contain internal septations. The presence of thickened septations (> 2 mm), nodules of enhancing soft tissue, rapidly growing painful lesions or lesions which are < 75% fat should raise concern for malignancy, although liposarcoma of the foot and ankle is rare [14, 17]. Signal change may occur in lipomas in weight-bearing locations secondary to inflammation, haemorrhage and infarction [14, 15]. When lipomas occur in muscle, muscle fibres traversing the lesion may mimic thickened septations and care must be taken not to confuse this with evidence of malignancy [14].

### Soft tissue chondroma

A benign extraosseous extrasynovial cartilage-forming tumour composed of mature hyaline cartilage which develops in soft tissue, unattached to bone [14, 15, 19]. There is a slight male preponderance and the majority of lesions occur in the hands and feet [14, 15, 19].

**Fig. 6:**
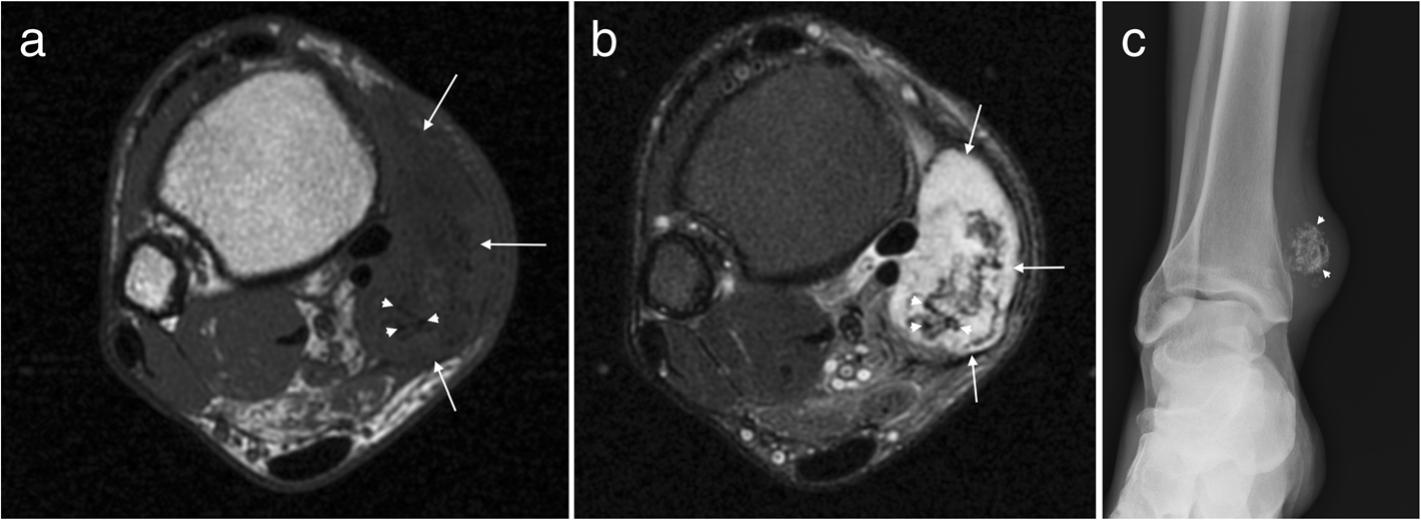
Soft tissue chondroma. Axial T1W (**a**) and T2FS (**b**) images of the ankle with a large well-defined posteromedial mass (arrows) which appears T1 hypointense and T2 hyperintense with internal low signal chondroid foci (arrowheads). The chondroid matrix is readily appreciated on the corresponding radiograph (**c**)

MRI demonstrates a well demarcated lobulated soft tissue mass isointense to cartilage, low to intermediate signal on T1W and hyperintense on T2W (Fig. 6). There may be central signal voids corresponding to mineralisation, which may be better appreciated on plain film radiography [17, 19]. Large lesions may be difficult to differentiate from extraosseous chondrosarcoma, which is an infrequent malignant tumour, and primary involvement of the foot is rare [13, 15].

### Pigmented villonodular synovitis and giant cell tumour of the tendon sheath

Pigmented villonodular synovitis (PVNS) is a benign proliferative intra-articular disorder of the synovium. Giant cell tumour (GCT) of the tendon sheath is the slightly more common extra-articular manifestation of the disorder which can affect bursae, tendons and ligaments [14–16]. They may occur at any age but favour the 3rd–4th decade [14–16]. Both giant cell tumour and PVNS are composed of multinucleated giant cells with intra- and extra-articular deposition of haemosiderin, macrophages, fibroblasts and xanthoma cells [1, 13]. The hypervascular nature of these lesions frequently results in haemorrhage. PVNS is usually mono-articular, and most often affects the ankle and tarsal joints presenting as painful joint swelling [16, 18]. GCT is more common in the forefoot and typically present as a painless slow growing mass [16, 17, 19].

**Fig. 7:**
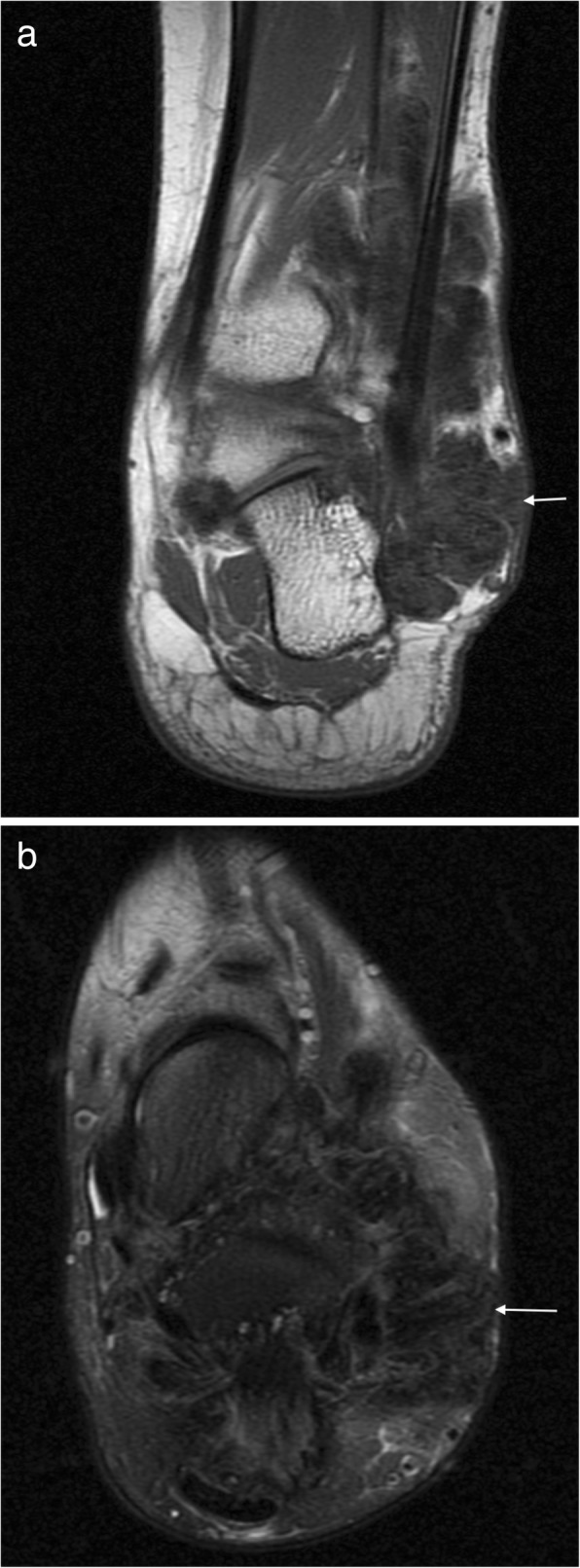
PVNS. Coronal T1 (**a**) and axial T2FS (**b**) images of the ankle demonstrating extensive abnormal soft tissue with intermediate signal on T1W and hypointense on T2FS

**Fig. 8:**
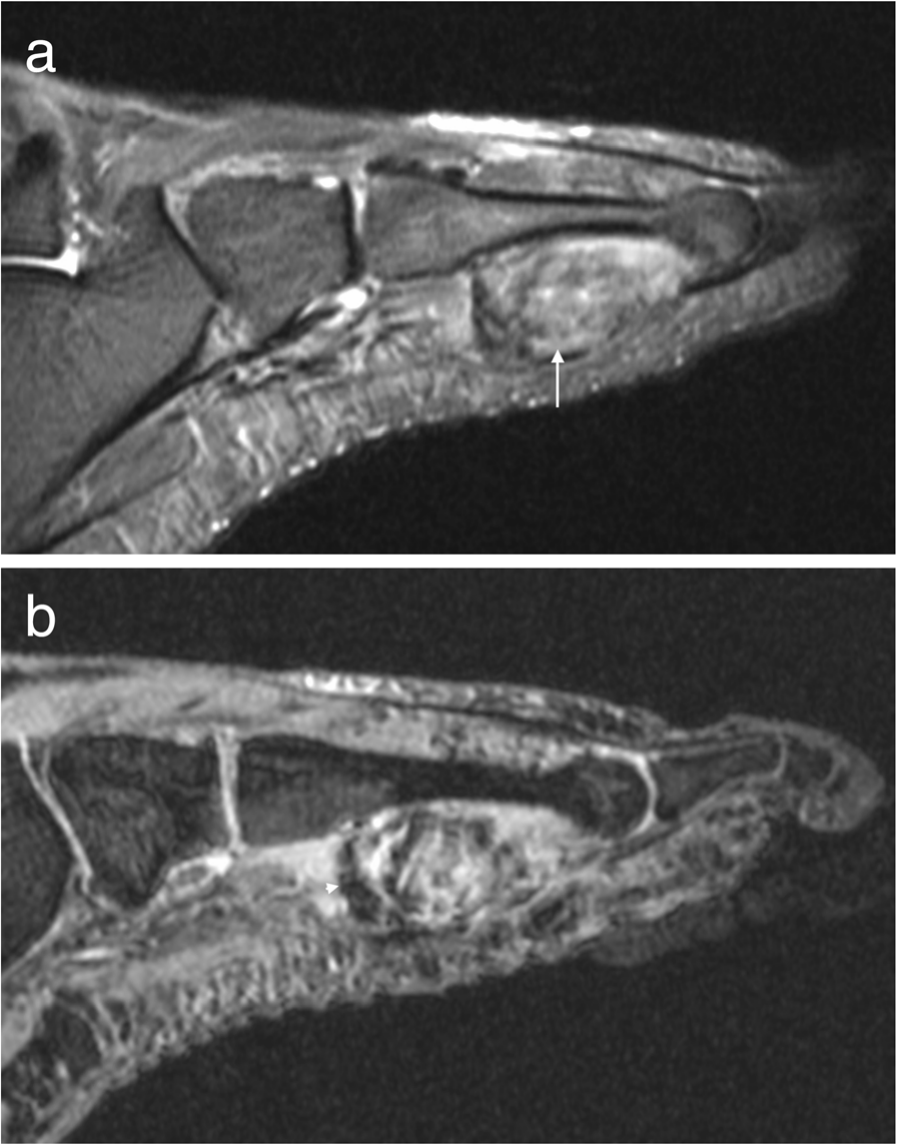
Giant cell tumour of the tendon sheath. **a** Sagittal T2FS image shows a heterogeneously hyperintense lesion underlying the metatarsal, related to the flexor tendon. **b** Gradient echo imaging shows foci of blooming (arrowhead)

PVNS does not generally contain mineralisation but may be visible as dense effusions on plain radiographs [16, 19]. T1W typically shows mixed intermediate to low signal soft tissue in the affected joint, depending on the relative proportion of fat, collagen and hemosiderin content (Fig. 7). There is a characteristic “blooming” of haemosiderin products on gradient-echo imaging; however, this is not specific for PVNS and the differential for this appearance should include haemophilia, amyloid and rheumatoid arthritis [16, 19]. The soft tissue mass demonstrates heterogeneous enhancement and there may be well-defined erosions and cysts with thin sclerotic margins on both sides of an affected joint [14]. GCT appears similar to PVNS but is related to a tendon and is more often homogenously hypointense with more homogeneous enhancement (Fig. 8) [1]. The main differential for GCT is a tendon sheath fibroma, which may have a similar appearance, but are less common and do not demonstrate “blooming” on gradient-echo sequences [16, 17].

### Synovial osteochondromatosis

Synovial osteochondromatosis is a disorder of the synovium resulting in metaplastic nodules of cartilaginous proliferation within joints, bursae or tendon sheaths which proceed to mineralise and detach [1]. It is twice as common in men as in women and most often presents in the 3rd–5th decade [14, 16, 19]. It is often mono-articular but may be bilateral [16]. Malignant transformation into secondary synovial chondrosarcoma is rare.

**Fig. 9:**
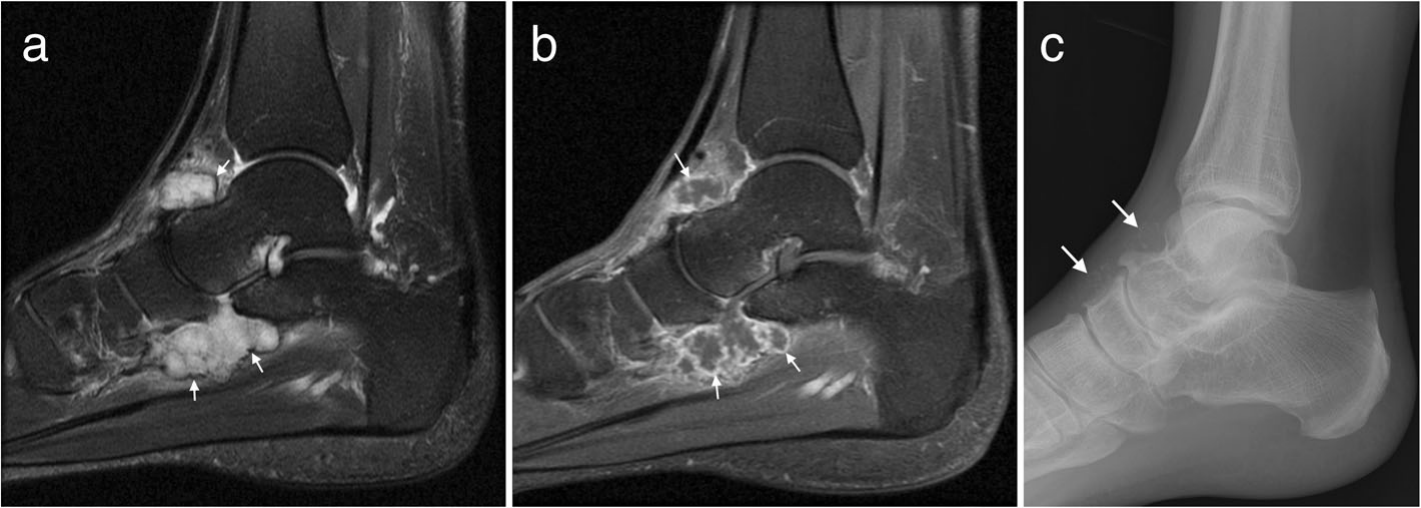
Synovial chondromatosis. **a** Sagittal T2FS image of the ankle demonstrating a lobulated T2 hyperintense lesion with a low signal rim (arrows) and **b** avid synovial enhancement on post contrast T1FS imaging (arrows). **c** Mineralisation is better appreciated on the corresponding radiograph (arrows).

In the late stage, plain radiographs or CT may demonstrate numerous intra-articular mineralised bodies with secondary osteoarthritis. Earlier in the disease, non-mineralised intra-articular bodies present a more challenging diagnosis. In the initial proliferative phase, a lobulated intra-articular lesion is typical, with intermediate to slightly hyperintense signal on T1W and high signal on T2W (Fig. 9) [14, 16, 19]. Later, mineralised nodules may be seen as signal voids on all sequences, or as ossified bodies with a low signal cortex and central fatty marrow signal [14, 16, 19]. Correlation with radiographic appearances can be helpful to further delineate and classify any foci of mineralisation. Post contrast imaging shows enhancement of the hyperplastic synovium, more pronounced in the earlier stage [14, 16].

### Peripheral nerve sheath tumours

Neurofibroma and schwannoma are relatively uncommon around the foot and ankle accounting for 5.4% and 3.9% respectively of all benign soft tissue lesions in this region [12]. They most often occur in the 3rd and 4th decade and often present as slow growing painless lumps, though neurological symptoms may be present [18, 20]. The majority are isolated lesions, but multiple or plexiform lesions may be seen in neurofibromatosis type I [16, 20].

**Fig. 10:**
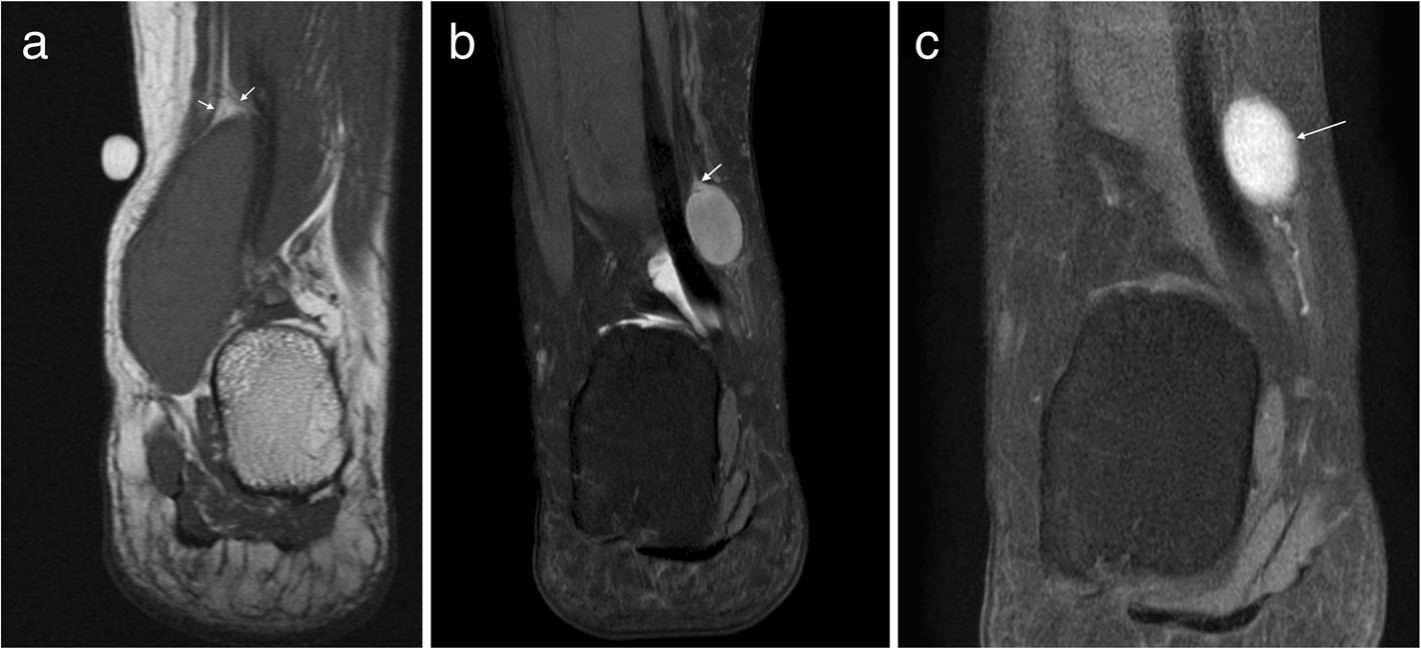
Schwannoma. **a** Coronal T1W image demonstrating a well-defined homogeneously T1 isointense lesion with the split fat sign (arrows). **b** Coronal T2FS image of another schwannoma showing a homogeneously hyperintense lesion with a “ball on a string” sign denoting origin from the posterior tibial nerve (arrow). **c** The same lesion demonstrates avid enhancement on the post-contrast T1FS image

These lesions are generally ovoid in shape, isointense to skeletal muscle on T1W and hyperintense on T2W with heterogeneous enhancement (Fig. 10) [16, 20, 27, 29]. Several classic MRI features are said to be suggestive of nerve sheath tumours [18, 20, 29]. The “split fat” sign describes a thin rim of fat encompassing the lesion, best seen on T1W. The nerve of origin may be seen entering and leaving the lesion, known as the “ball on a string” sign. The “target sign” describes peripheral high signal with central low signal on a T2W cross section of the lesion, and is more common in neurofibromas. However, this is non-specific and may also be seen in both schwannoma and malignant nerve sheath tumours [20]. Schwannomas are generally eccentrically located and separable from the nerve at surgery, whereas neurofibromas tend to infiltrate the nerve necessitating nerve sacrifice at resection [18, 20].

## Malignant lesions

### Mesenchymal lesions

#### Dermatofibrosarcoma protuberans

A rare slow growing superficial mass which may ulcerate, most often seen in adolescents [20, 30]. It demonstrates iso- to slightly hypointense signal on T1W and high signal on both conventional T2W and fat suppressed T2W sequences with intense enhancement [20, 27, 30]. Increased T2 signal may be seen spreading from the lesion within the adjacent skin [20]. Dermatofibrosarcoma protuberans (DFSP) has a low metastatic potential but recurrence after surgical resection may occur [20, 30]. This is partly attributable to protrusions of tumour into the subcutaneous fat (giving rise to the “protruberans” part of the name).

#### Synovial sarcoma

Synovial sarcoma is the fourth most common soft tissue sarcoma, 18–25% of which occur in the ankle and foot making it the most common malignancy in this region [14–16, 31]. Despite its name, it is in fact a lesion of mesenchymal origin and is named for its histological resemblance to synovial tissue [16, 31]. It occurs in young adults, typically between 15 and 45 years of age [14, 16, 19]. It is typically related to tendon sheaths, bursae and less frequently aponeuroses, fascia and ligaments [15, 31]. There is no relationship with synovial tissue and an intra-articular location is very rare; however, 75% occur within 5 cm of a joint [14, 15, 31]. There are three main histological subtypes: monophasic, biphasic and poorly differentiated, 90% of which stain positive for keratin [20, 31]. Cytogenetic analysis may reveal a t(X;18) translocation [20, 31].

Patients typically present with a slow growing palpable mass with an average duration of symptoms of 2–4 years [31]. Unlike other sarcomas, pain and tenderness are common findings [31]. Unfortunately, the overall prognosis is guarded with recurrence following surgical resection seen in approximately 50% cases and metastases to lungs, bones or regional nodes in approximately 40% [19, 31].

**Fig. 11:**
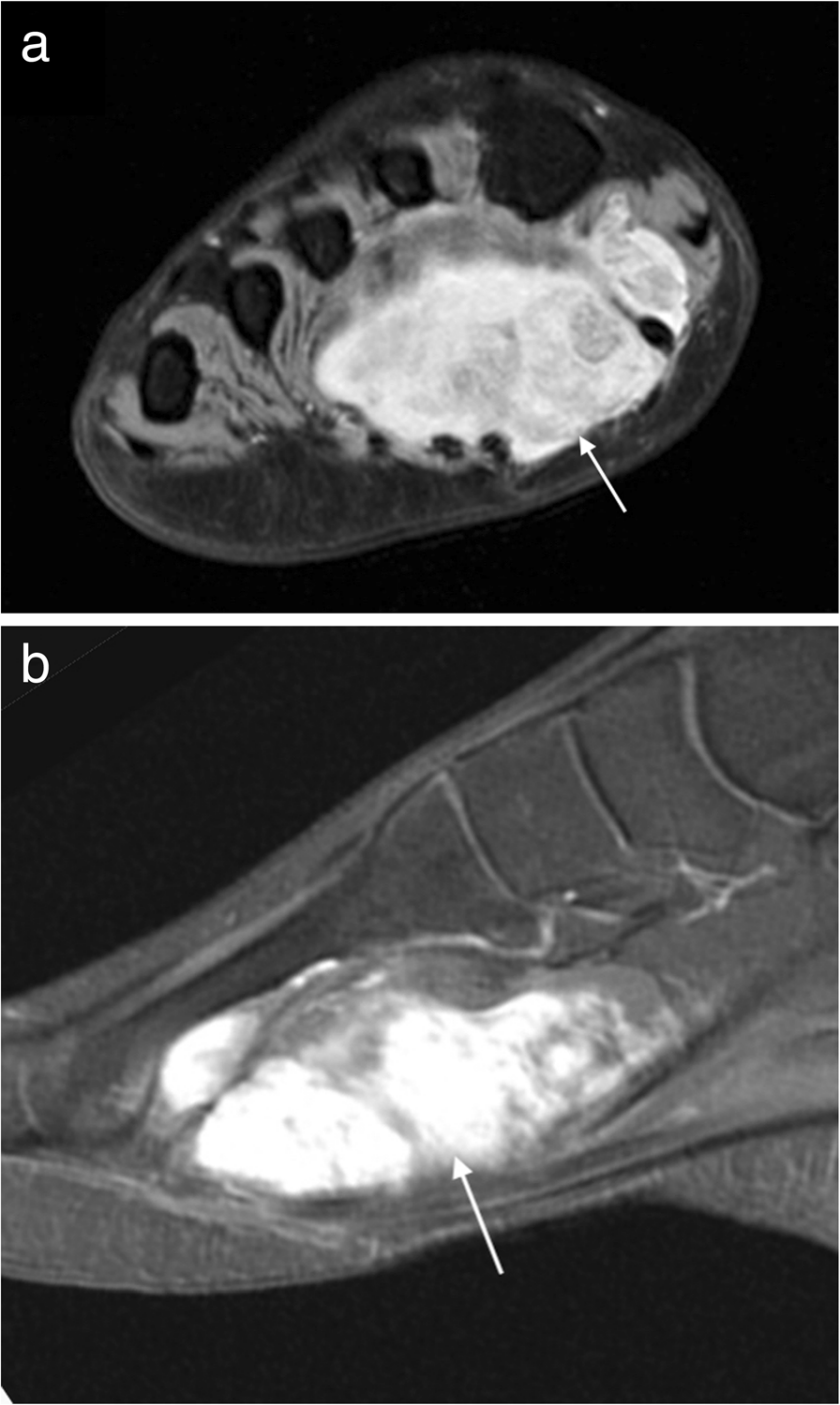
Synovial sarcoma. Short axis T2FS image of the foot (**a**) showing a heterogeneous T2 hyperintense mass on the plantar aspect of the foot with heterogeneous enhancement of the viable tumour on the sagittal post-contrast T1FS image (**b**)

The MRI appearance is variable depending on size and rate of growth but is typically a T1 hypointense and T2 hyperintense mass with enhancement of viable tumour on post contrast imaging (Fig. 11) [14, 15, 20, 31]. Slow growing masses are well defined, whereas rapidly growing lesions tend to have a more infiltrative appearance [14, 15, 31]. Smaller tumours may have the appearance of simple cysts and attempted aspiration of these lesions can lead to disruption of the capsule and compromise of the subsequent surgical resection bed [20, 31]. Whenever a cystic-appearing lesion is identified in an atypical location, post contrast imaging should be performed to exclude a more sinister lesion.

Larger lesions may contain solid, cystic, necrotic and haemorrhagic components and will therefore appear heterogeneous on all sequences and may demonstrate the “triple sign” (low, intermediate and high signal within the lesion on T2W sequences) [14, 20, 31]. Septations within a large lesion can give the classic “bowl of grapes” appearance [20, 31]. Lesions arising within muscle may demonstrate a “split fat” sign, although this is true of any intramuscular lesion [31]. Fluid-fluid levels are present in 10–25% cases [19]. Around 20–30% demonstrate calcification, often better seen on plain radiographs or CT [14, 15, 20, 31]. Erosion of the adjacent bone may occur and given the slow growth, often has an indolent appearance which may give the false impression of a benign lesion, particularly on plain radiography [16, 19, 31].

#### Rhabdomyosarcoma

Rhabdomyosarcoma is a primitive malignant soft tissue sarcoma with a skeletal muscle phenotype [32]. There are three main subgroups: embryonal, alveolar and pleomorphic. Embryonal tends to occur in the first decade of life, whereas alveolar type is more common in adolescents and young adults [33, 34]. Metastases occur to the lymph nodes, lungs and bones [34]. The 5-year survival is estimated at 27% in adults and 61% in children [34].

**Fig. 12:**
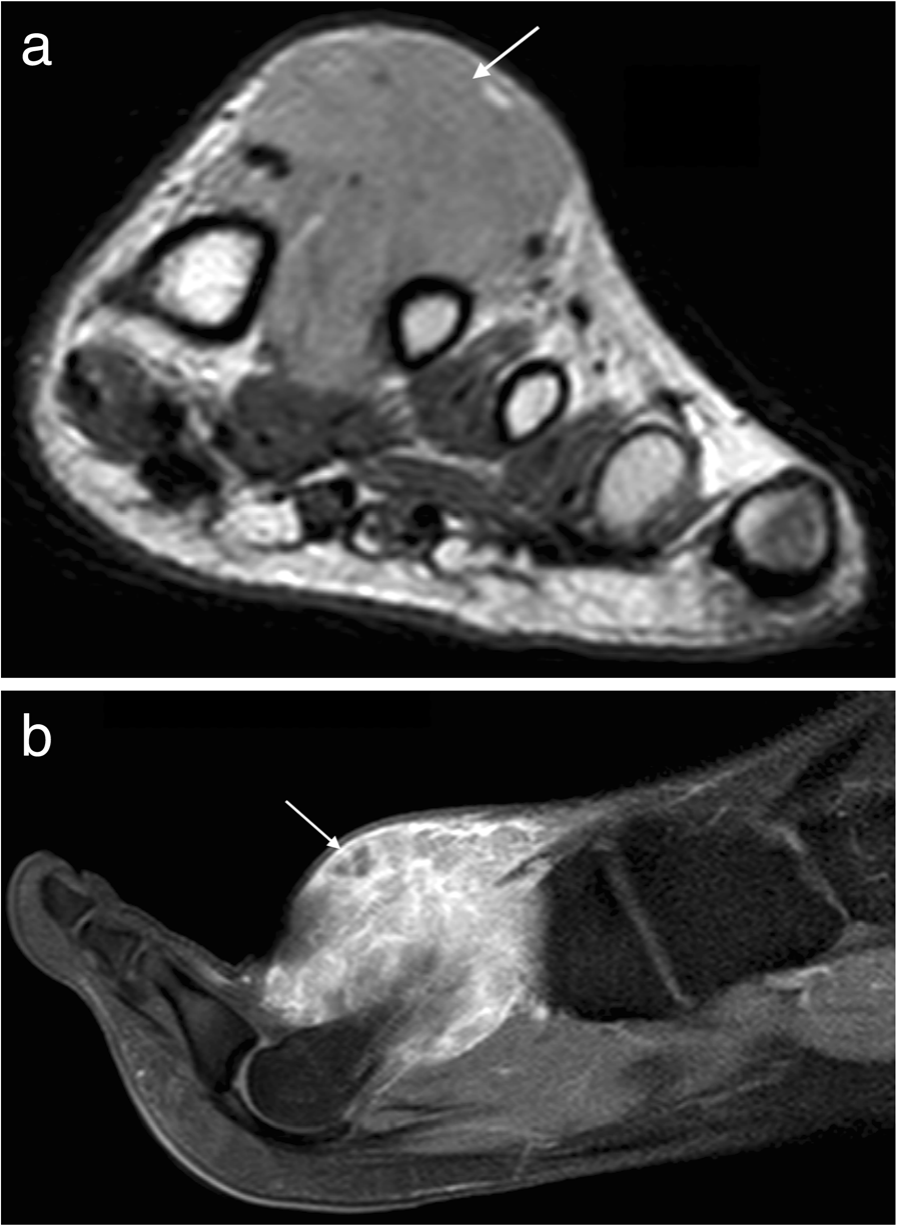
Rhabdomyosarcoma. Short axis T1 image (**a**) of the foot of a 14-year-old girl demonstrating a hyperintense mass in the first web-space with avid enhancement on postcontrast T1FS imaging (**b**)

Alveolar and pleomorphic tumours are usually large necrotic lesions with a lobulated margin and lymphovascular invasion [34]. MRI demonstrates a lesion that is iso- to hyperintense to muscle on T1W, heterogeneously hyperintense on T2W with moderate to marked enhancement with contrast (Fig. 12) [33]. Prominent serpentine flow voids are often a feature of these highly vascular lesions, as is internal haemorrhage [33].

#### Malignant peripheral nerve sheath tumours

These rare aggressive neoplasms are most often seen in the context of neurofibromatosis type 1 [35]. They can be difficult or impossible to distinguish from benign peripheral nerve sheath tumours on imaging alone. Malignant lesions tend to be larger (> 5 cm) and rapid growth is often the only clinical evidence of malignant behaviour, though this is neither a sensitive nor a specific finding [17, 35]. They have a less well-defined margin with peritumoural oedema [17, 35]. In addition, intra-tumoural lobulation and destruction of adjacent bone are suggestive of malignancy [35]. A recent study by Ahlawat utilising DWI demonstrated that the presence of a “target sign” (peripheral hyperintensity and homogeneous central hypointensity) on high *B* value sequences and the ADC map is highly suggestive of a benign lesion [36].

#### Other sarcomas

**Fig. 13:**
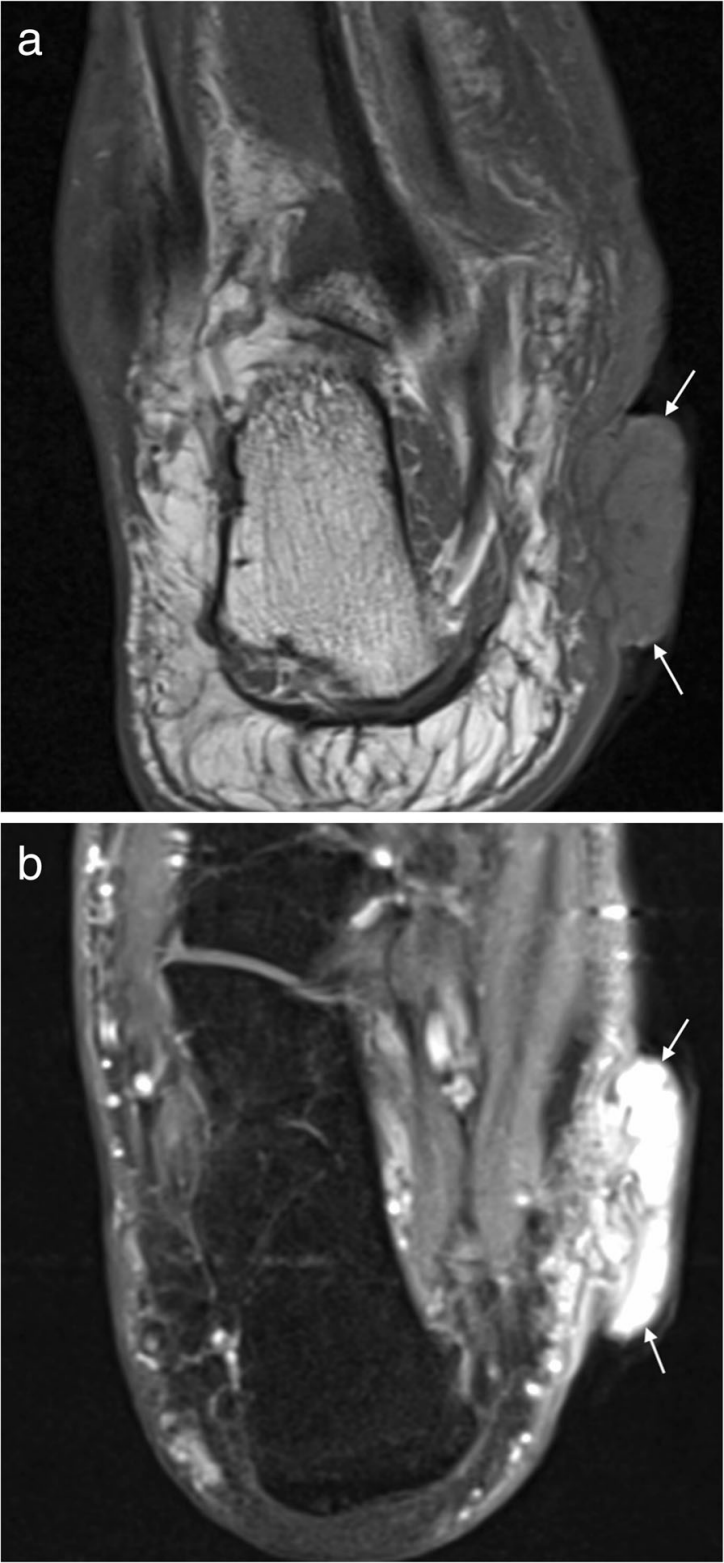
Kaposi’s sarcoma. Coronal T1W (**a**) and post-contrast T1FS (**b**) images of the foot showing an exophytic lesion, low signal on T1W with avid enhancement and a nodular proliferation of the dermis

Kaposi sarcoma is a rare malignancy associated with human herpes virus 8 (HHV-8). There are four subtypes: AIDS-related Kaposi sarcoma (KS), immunocompromised KS, endemic African KS and classic KS which is most commonly found in elderly Jewish and Mediterranean. In the lower limbs, it is often associated with venous stasis and lymphoedema and classically appears as a pedunculated violaceous lesion [37]. At MRI, it may appear as an exophytic and pedunculated lesion, low signal on T1W and high signal on T2W with avid enhancement and a nodular proliferation of the dermis (Fig. 13).

**Fig. 14:**
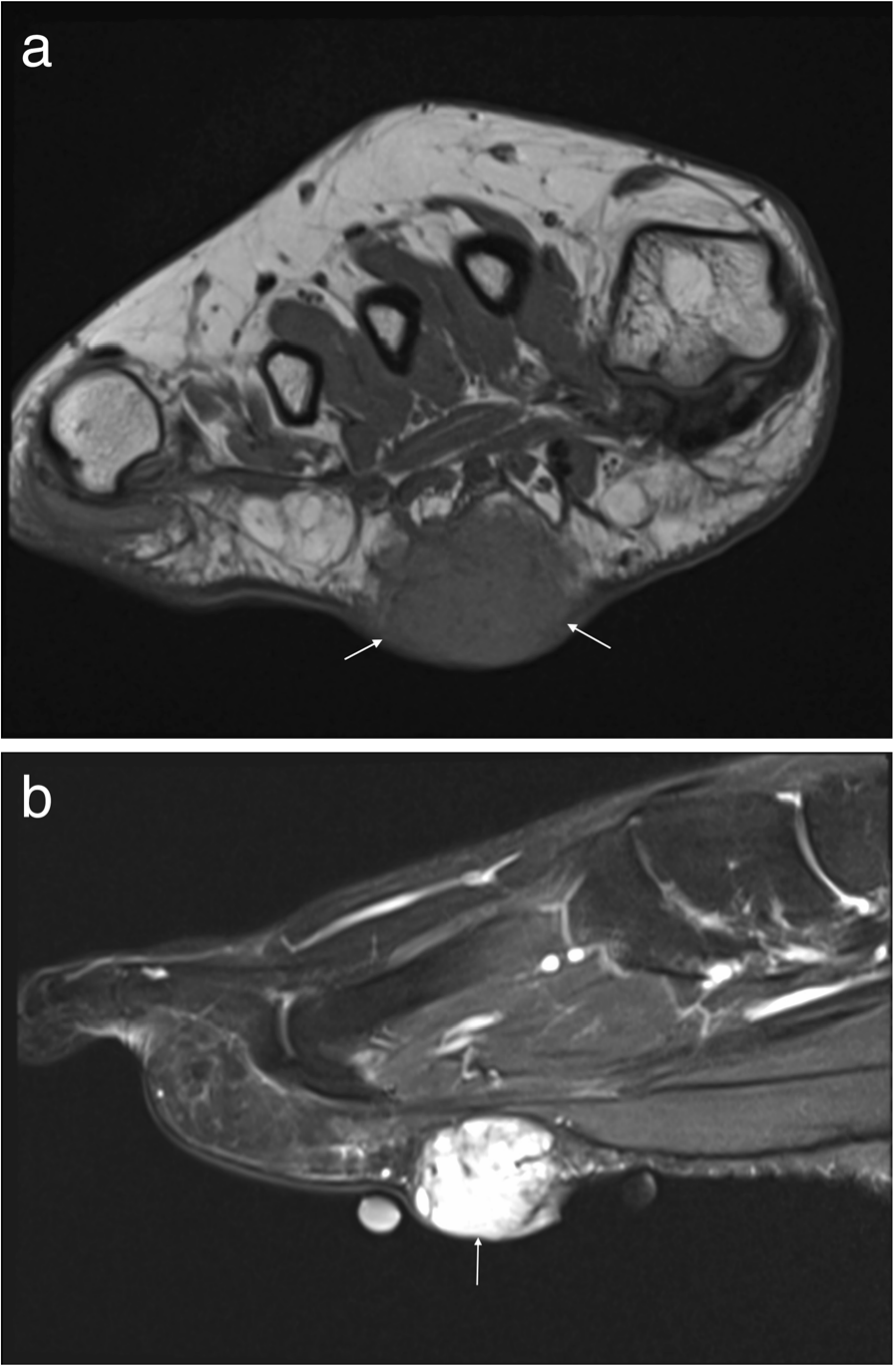
Extraosseous Ewing’s sarcoma. **a** Coronal T1W and (**b**) sagittal T2FS images of the foot. The lesion is isointense on T1W and hyperintense on T2FS and lies superficial to the plantar fascia

Other sarcomas which may occur in this region include clear cell sarcoma, undifferentiated pleomorphic sarcoma (formerly malignant fibrous histiocytoma), extra-osseous Ewing’s sarcoma (Fig. 14), leiomyosarcoma, myxofibrosarcoma, angiosarcoma and liposarcoma. In some of these lesions, certain findings may be suggestive of an underlying pathology. Clear cell sarcoma may be T1 hyperintense in up to 52% cases due to melanocytic content [38]. Well-defined liposarcomas will contain fat. Myxoid liposarcoma and myxofibrosarcoma contain myxoid matrix, with characteristic T2 hyperintensity and diffuse mild to moderate enhancement, or thickened rim enhancement with internal septae [39].

### Lymphoma

**Fig. 15:**
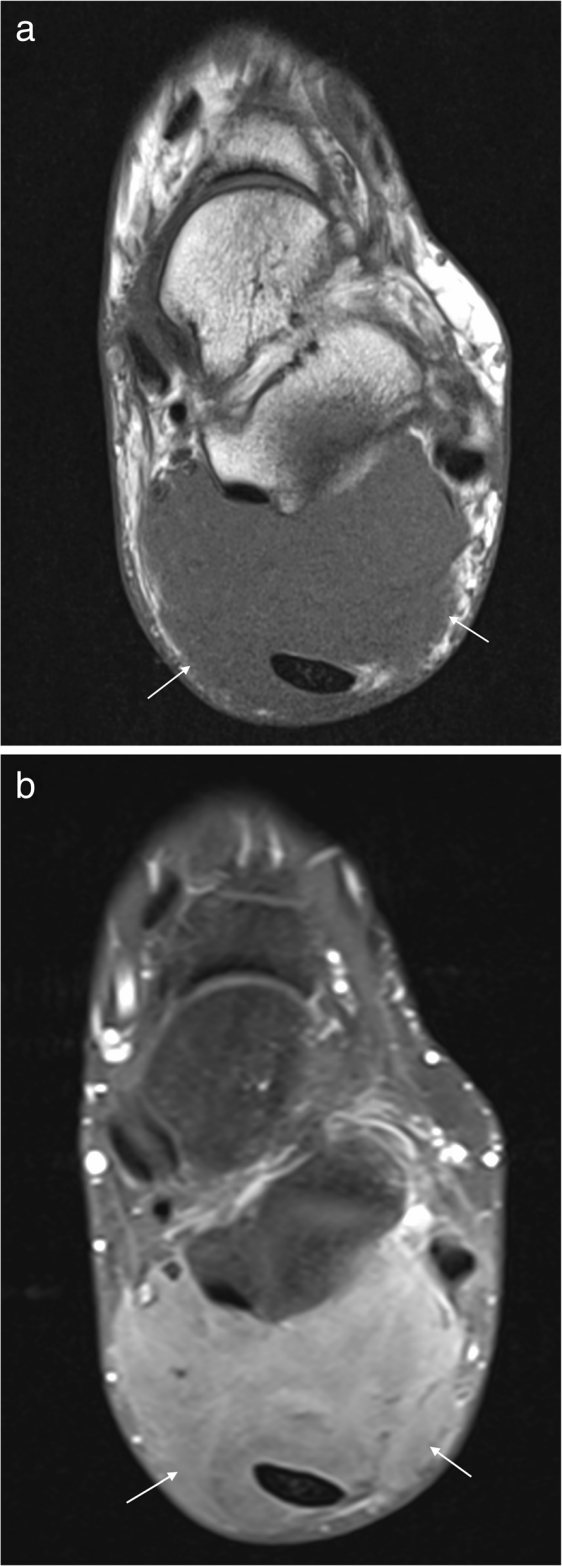
Lymphoma. Axial T1FS (**a**) and post-contrast T1FS (**b**) images of the ankle demonstrating an infiltrative process surrounding the posterior tendons and neurovascular structures, isointense to skeletal muscle on T1W with mild diffuse contrast enhancement

Primary soft tissue lymphoma is extremely rare and while secondary involvement of soft tissues is more commonly encountered, this too is a rare occurrence. On MRI, the lesion appears isointense to skeletal muscle on T1W and intermediate to high signal on T2W with mild diffuse contrast enhancement (Fig. 15) [40]. Areas of internal necrosis or haemorrhage are not generally a feature. Infiltration of the adjacent subcutaneous tissues may mimic an inflammatory process [40]. Regional lymph nodes should also be evaluated for disease involvement. Rarely a primary cutaneous lymphoma (e.g. mantle cell lymphoma) may affect the foot presenting as a single or multiple non-healing ulcers [41].

### Melanoma

Cutaneous melanoma is most often a clinical diagnosis but may occasionally be seen on MRI, occasionally to assess for deep invasion. Pigmented lesions have a characteristic high signal on T1W but less pigmented lesions will have lower T1 signal [20]. They are hyperintense on T2W and enhance avidly with contrast [20]. Multiple lesions may be present in metastatic disease [20].

### Squamous cell carcinoma

Squamous metaplasia in the setting of chronic ulceration may lead to malignant transformation. A non-healing ulcer or persistent draining sinus should raise suspicion for malignancy (known as a Marjolin’s ulcer) [20]. MRI features are non-specific but the diagnosis should be considered in the presence of an enhancing soft tissue mass adjacent to a site of chronic osteomyelitis or ulceration [20].

## Summary

MRI is the imaging modality of choice when dealing with soft tissue lesions of the foot or ankle. Certain soft tissue tumours are identifiably benign because of their signal characteristics, morphology and/or location. These include plantar fibromatosis, haemangioma, lipoma, PVNS/GCT tendon sheath and synovial chondromatosis. Sometimes, soft tissue chondroma and peripheral nerve sheath tumours will be relatively characteristic in appearance. For other non-specific soft tissue masses, a high level of suspicion for malignancy and a low threshold for obtaining tissue should be maintained.

A lesion lacking features of a specific benign entity should be regarded as malignant until proven otherwise. Features that are more common in malignant than in benign lesions include large size (> 5 cm), deep site, inhomogeneous signal intensity, haemorrhage and necrosis, early and inhomogeneous contrast enhancement, irregular margins, surrounding soft tissue oedema and invasion of adjacent structures, including bone and neurovascular structures [17]. Advanced MRI techniques such as spectroscopy, perfusion and diffusion-weighted imaging may contribute to better soft tissue characterisation.

Referral for biopsy is indicated when there is a reasonable suspicion of malignancy, in any superficial lesion > 5 cm, any deep lesion regardless of size and in indeterminate cases. Biopsy should be planned and performed in a regional sarcoma referral centre, involving multidisciplinary input, including that of an oncologic surgeon. Core biopsy is required as fine needle aspiration is inadequate. The approach should be planned to avoid traversing non-affected compartments and the biopsy tract removed en bloc at the time of surgery. MRI, US and FDG PET may be used to guide biopsy to sites of viable tumour, avoiding sites of necrosis or haemorrhage.

A comprehensive knowledge of the imaging characteristics of the soft tissue lesions which occur around the foot and ankle will enable a radiologist to confidently identify those lesions which have a characteristic imaging appearance and, most importantly, to identify those lesions which require further imaging or surgical referral.

## Abbreviations

ADC: Apparent diffusion coefficient
CT: Computed tomography
DCE: Dynamic contrast-enhanced
DFSP: Dermatofibrosarcoma protuberans
DWI: Diffusion-weighted imaging
FDG PET/CT: Fluorine-deoxyglucose positron emission tomography/computed tomography
FS: Fat suppression
GCT: Giant cell tumour
ISSVA: International Society for the Study of Vascular Anomalies
KS: Kaposi sarcoma
MRI: Magnetic resonance imaging
PD: Proton density
PMRS: Proton magnetic resonance spectroscopy
PVNS: Pigmented villonodular synovitis
SUV: Standardised uptake value
SWI: Susceptibility weighted imaging
T1W: T1-weighted
T2W: T2-weighted
US: Ultrasound
